# Nanoencapsulation of n-butanol extract of *Symphytum kurdicum* and *Symphytum asperrimum*: Focus on phytochemical analysis, anti-oxidant and antibacterial activity 

**DOI:** 10.22038/IJBMS.2022.62032.13760

**Published:** 2022-03

**Authors:** Elaheh Mahmoudzadeh, Hossein Nazemiyeh, Hadi Valizadeh, Farnaz Khaleseh, Samin Mohammadi, Sanaz Hamedeyazdan

**Affiliations:** 1 Faculty of Pharmacy, Tabriz University of Medical Sciences, Tabriz, Iran; 2 Department of Pharmacognosy, Faculty of Pharmacy, Tabriz University of Medical Sciences, Tabriz, Iran; 3 Research Centre for Pharmaceutical Nanotechnology, Tabriz University of Medical Sciences, Tabriz, Iran; 4 Research Center for Pharmaceutical Nanotechnology, and Department of Pharmaceutics, Faculty of Pharmacy, Tabriz University of Medical Sciences, Tabriz, Iran; 5 Department of Pharmaceutics, Faculty of Pharmacy, Tabriz University of Medical Sciences, Tabriz, Iran; 6 Pharmaceutical Sciences Research Center, Health Institute and School of Pharmacy, Kermanshah University of Medical Sciences, Kermanshah, Iran; 7 Research Center for Integrative Medicine in Aging, Aging Research Institute, Tabriz University of Medical Sciences, Tabriz, Iran

**Keywords:** Anti-oxidants, Boraginaceae, Comfrey, Flavonoids, Liposome, Microbial sensitivity tests, Nanoparticle

## Abstract

**Objective(s):**

The current study’s objectives were to obtain different extracts and essential oils of *Symphytum kurdicum* and *Symphytum asperrimum* and to determine the chemical composition, as well as to evaluate free radical scavenging activity (IC_50_) and minimum bactericidal concentration (MBC), and the effect of liposomal formulation on antimicrobial properties.

**Materials and Methods:**

Air-dried powdered aerial parts of *S. kurdicum* and *S. asperrimum* were used. The antioxidant and antibacterial properties, essential oil compositions, total phenol, and flavonoid contents of different fractions were determined by DPPH test, disk diffusion assay, gas chromatography-mass spectrometry, Folin-ciocalteu reagent, and colorimetric assay method, respectively. The film hydration method was used to fabricate nanoparticles.

**Results:**

GC-MS analysis indicated that hexafarnesyl acetone was a major essential oil component. n-butanol and ethyl acetate extracts of *S. kurdicum* had the highest anti-oxidant activity. Extracts of both plants showed antimicrobial activity. The extracts’ maximum inhibition zones against *Staphylococcus epidermidis* were established. A particle size analyzer detected the formulation size of 140 nm. The optimum formulation of liposomes contains the ratio of 75 mg lecithin, 25 mg cholesterol, and 50 mg herbal extract. Despite the nanoparticles’ appropriate particle size, the liposomal extract’s antimicrobial effect was lower than that of the free form.

**Conclusion:**

Our findings demonstrated that extracts have significant antibacterial and anti-oxidant activities, attributed to their bioactive constituents.

## Introduction

In recent years, pharmacophores acquired from herbals have had an important role in medication discovery. Additionally, they were used for developing antibiotic-resistant microbial strains in search of novel antimicrobial agents (1). Frequently, there is a correlation between the polyphenolic content of herbs and the antimicrobial or anti-oxidant effects of plant extract preparations, which could help in the identification of the active ingredients (2). This genus is a member of the *Boraginaceae* family (3). These herbaceous plants are characterized mainly by large, hairy leaves, and tuberous roots (4). 

The essential oil yield of *Boraginaceae* members is low (5, 6). According to the most recent research, plants in the *Symphytum* genus have numerous pharmacological effects, including antimicrobial, anti-oxidant, and anti-inflammatory properties. For example, in a study by Rocha *et al*., *Symphytum officinale L. *had a significant antifungal effect against *Sclerotinia sclerotiorum *(7, 8). An *in vitro* study revealed that *S. officinale L. *extracts have antimicrobial activity against various bacterial strains, particularly *Staphylococcus aureus* (9). It could be attributed to the aqueous extract’s phenolic content (allantoin, rosmarinic acid, caffeic, and chlorogenic acids) (10, 11). 

Researchers investigated various carriers for herbal extracts loading, such as manganese nanoparticles and micelles (12, 13). Liposomes are some of the most interesting; additional studies have confirmed the effect of liposomal structures on herbal extract efficacy. The spherical form of liposomes composed of phospholipids provides the situation for loading both hydrophilic and lipophilic components that, in addition to biocompatibility and non-immunogenic properties, makes it a good choice for loading herbal extracts. Soybean lecithin is a natural phospholipid for preparation of liposomes with acceptable safety. As studied by Singh *et al*. liposomal neem (*Azadirachta indica*) gel composed of soybean lecithin and cholesterol demonstrated *in vitro* drug diffusion and skin retention equal to 62.178% ± 0.91 and 20.03% ± 0.63, respectively (14). Researchers studied the encapsulation of *Laurus nobilis* leaf extract in liposomes. The results showed that liposomal encapsulation improved the antimicrobial and anti-oxidant properties of the extract compared with non-encapsulated. They also confirmed the application of nano-formulation as a natural preservative for meat products (15).

The genus *Symphytum* produces allantoin, phenolic acids, and flavonoids (5) whose antibacterial and anti-oxidant properties have been recognized (16). The main distribution centers of the genus *Symphytum*, with 113 species, are humid meadow and lake edge parts of Asia, Europe, and America (3). Most phytochemical and biological studies have been conducted on these species. Different *Symphytum* species have various therapeutic applications (16). In the present study, we focused on native Iranian medicinal plants,* Symphytum* species,* S. asperrimum *and* S. kurdicum*, the biological properties and phytochemicals of which are unknown. To the best of our knowledge, no research has been published on the two mentioned plants yet. The current study aimed to evaluate different extracts and essential oils in various aspects. The extracts were assessed for phytochemical characteristics, as well as potential antimicrobial and anti-oxidant activities. The other goal of the study was to fabricate the preparation of liposomal formulation of extracts and assess the effect of encapsulation on the antibacterial properties of the extract. 

## Materials and Methods

***Chemicals*** 

Analytical grade solvents containing n-hexane, chloroform, ethanol, ethyl acetate, *n*-butanol, and methanol were obtained from Dr. Mojallali (Iran). Folin-Ciocalteu, gallic acid, aluminum chloride, sodium bicarbonate, sodium acetate, and dimethyl sulfoxide (DMSO) were acquired from Merck (Germany). 2, 2-diphenyl-1-picrylhydrazyl (DPPH) and quercetin were acquired from Sigma-Aldrich (Germany). Mueller-Hinton agar and Mueller-Hinton broth were obtained from Quelab (Canada). Amikacin and Tetracycline antimicrobial susceptibility test disks were purchased from Padtan Teb (Iran). 

***Plant materials, solvent extractions, and fractionations***

Aerial parts of *S. kurdicum* and *S. asperrimum* were collected from ghasemlou valleys, West Azerbaijan province, and Arasbaran forest, East Azerbaijan province, (Iran May 2019), respectively. The specimens were identified and deposited in the Herbarium of the Faculty of Pharmacy, Tabriz Medical University, Iran (TBZFPH (No. 4072)) and (TBZFPH (No. 4073)). Aerial parts of the collected plants were separately air-dried, powdered, and subjected to soxhlet extraction. *n*-Hexane and chloroform solvents were used in the extraction process using soxhlet apparatus individually, and then the extraction process continued with the maceration technique. The subsequent solvent was ethanol: water (70:30 V:V). The obtained hydroalcoholic extracts were dried, dispersed in water, then fractionated, initially decanted with ethyl acetate and later with *n*-butanol. The yielded extracts were dried at low temperature under vacuum via a rotary evaporator (Heidolph, Germany). Dried extracts were stored at -8 °C until analysis. 

***Extraction of essential oil***

Hydrodistillation was conducted separately for both plants using a Clevenger apparatus to extract the essential oil. One hundred grams of air-dried plant materials were weighed into a round 1000 ml flask. Then, 750 ml of distilled water and some glycerol were added to the flask. Extraction was performed for two hours. Ultimately, the essential oils were collected and placed into vials and stored at 4 °C for later analysis.

***Gas chromatography- Mass (GC/MS) analysis***

The components of the essential oils were assessed using a Shimadzu GCMS-QP5050A equipped with a DB-1 column (60 m × 0.25 mm; film thickness 0.25 ϻm). The split ratio was 1: 24. The primary oven temperature was 50 °C which was kept for 3 min then raised to 270 °C at a rate of 4 °C/min. As a final point, the predicted component’s RI values (retention index) were calculated and compared with the valid RI values from the NIST 2018 library. Additionally, the qualified percentage of each compound was reported according to the area under the curve (17).

***Antimicrobial activity***

A disk diffusion assay was used to examine the antimicrobial activities of *S. kurdicum* and *S. asperrimum*. Two gram-positive and two Gram-negative bacteria were used as the test organisms: *Staphylococcus aureus* Persian Type Culture Collection (PTCC 1337), *Staphylococcus epidermidis* (PTCC 1435), *Escherichia coli* (PTCC 1330), *Salmonella typhi* (PTCC 1074), and a fungal strain, *Candida albicans* (PTCC 5027). Different extracts were suspended in DMSO solvent; bacterial suspensions were cultivated on the Mueller-Hinton agar medium (0.5 McFarland standard (108 CFU/ml)). 30 ϻl of the extracts were transferred to the blank disks. The standard antibiotic discs and negative control were tetracycline (30 µg per disc), amikacin (30 µg per disc), and DMSO (30 µl per disc), respectively. The average diameter of inhibition zones was recorded after 24 hr of incubation at 37 °C. Furthermore, the extracts’ minimum bactericidal concentrations (MBCs) were measured. The microbial concentration in wells was 5*105 CFU/ml, and the concentration of extracts varied from 0.39 mg/ml to 25 mg/ml. After incubation of the 96-well plates at 37 °C for 24 hr, culturing on the Mueller-Hinton agar plates was done to assay the minimum concentrations that could inhibit fungal or bacterial growth (18).

***Anti-oxidant activity***

Among all extracts, the *n*-butanol and ethyl acetate extracts were assessed for anti-oxidant ability using the DPPH method with some modification. Briefly, the extracts were dissolved in methanol (1 mg/ml) and serially diluted. Then, the DPPH solution was added to all samples in the 1:1 ratio. Consequently, the samples were shaken and incubated at room temperature for 30 min in the dark, and then the mixture was placed in a 10 mm micro cuvette. The samples’ absorbance was determined at 517 nm using a Spectronic Genesys 5 spectrophotometer. Inhibition percentages and average inhibition percentages were calculated to discover the correlation between the extract concentrations and average inhibition percentages. Subsequently, the concentrations which may reduce free radicals up to 50% (RC_50_) were measured according to the line balances (19). 

***Total phenol contents***

Total phenol content was acquired using the Folin-ciocalteu reagent. In the first instance, standard solutions of methanol and ethyl acetate extracts were prepared (0.1 mg/ml), then extract solutions (1 ml), Folin-ciocalteu reagent (200 ϻl), and sodium bicarbonate 2% solution (1 ml) were mixed in the test tubes. Test tubes were centrifuged for 5 min at 16128 G-force and incubated in the darkness for 30 min at 25 °C. The spectrophotometric absorbance of samples was measured at 750 nm. Moreover, gallic acid solutions in the 1-10 mg/ml concentration range were treated as samples to get the standard line equivalence (20).

***Total flavonoid contents***

Total flavonoid content was acquired using an aluminum chloride colorimetric assay reagent. Ten milligram of *n*-butanol and ethyl acetate extracts were dissolved in 10 ml of methanol (80%), then 2 ml of samples were added to the mixture of distilled water (400 µl) and the reagent (1 ml), which is formed of sodium acetate (49 M) and aluminum chloride (10 M). The absorbance values of arranged mixtures were determined after 20 min at 410 nm, using the spectrophotometer apparatus. Quercetin was the standard compound in the process. 5, 10, 15, 20, and 25 µg/ml concentrations of quercetin were prepared and assessed in the same way. As a final point, the total flavonoid content of the extracts was reported as a weight ratio percentage (quercetin equivalent: extract) (20). 

***Preparation of liposomal formulation***

The fabrication method of the liposomal formulation was the film hydration method, according to Plangsombat *et al*., with some modification (21). Therefore, different ratios of soybean lecithin, cholesterol, and sample extracts of *S. asperrimum *and* S. kurdicum*, were dissolved in 8 ml of chloroform to form a clear solution (Table 5). Evaporation of chloroform was performed under vacuum by rotary evaporator (Rotavapor R-215, Buchi-Switzerland). Removing the organic solvent would lead to a homogeny film at the bottom of the round bottom flask. For removing the different molecules of chloroform, N_2_ gas was applied. The lipid film was hydrated by water at the temperature of 70 °C, and for better hydration with fine particles, bath sonicator (Bandelin, Berlin, Germany) followed by probe sonicator (Bandelin, Germany) were utilized at 70 °C. The un-entrapped extract was precipitated by centrifugation at 4032 G-force (Eppendorf, Centrifuge 5810R, Hamburg, Germany), and for size reduction, the formulation was filtered through preheated 0.45 and then 0.22 filters (22). The formulation was freeze-dried to be dissolved in the proper amount of water with desired concentration for the antimicrobial test. A particle size analyzer evaluated the liposome’s size (Wing SOLD 2101, Shimadzu, Japan). The formulation was diluted using distilled water by the ratio of 10:1 to be detected by the instrument. 

## Results

***Essential oil analysis***

GC-MS analysis of the essential oils resulted in identification of 20 components for *S. kurdicum,* as shown in Table 1. A total of 95.62% of the *S. kurdicum* essential oils were identified, and any components found at 0.1% or less were not considered for identification. Hexahydrofarensyl acetone (34.2%), phytol (33.4%), n-hentriacontane (11.4%), and dibutyl phthalate (3%) were the main components of this essential oil. The other significant compounds were β-turmerone (1.4%), 6, 10, 14-trimethylpentadecane-2-ol (1.4%), and β-ionene (1.01%).

GC-MS analysis of *S. asperrimum* essential oil identified 8 components, as shown in Table 2. The main compounds of the essential oil were hentriacontane (31.03%), hexafarnesyl acetone (24.14%), heptane (10.3%), and phytol (6.03%).

***Total phenol and flavonoid contents***

Total phenolic and flavonoids contents of *n*-butanol and ethyl acetate extracts of *S. asperrimum* and *S. kurdicum* collected from North-West of Iran were presented in Table 3. The results showed variations in total phenolic and flavonoid contents, even in the same parts of the two genera of *Symphytum* species. *n*-butanol extract of *S. asperrimum* had the highest total phenolic (57.3 mg GAE/g extract) and flavonoids contents (31.78 mg QE/g extract). While *n*-butanol extract of *S. kurdicum* contained less total phenolic (42.59 mg GAE/g extract) and flavonoids contents (20.55 mg QE/g extract) than* S. asperrimum*.

***Anti-oxidant activity***

In order to determine the anti-oxidant properties of the extracts of the two plants, the DPPH (2, 2-diphenyl-1-picrylhydrazyl) method, a widely used method for determining the scavenging activities of various natural components, was used. It was utilized to measure the *in vitro* anti-oxidant properties of the extracts of *S. asperrimum* and *S. kurdicum*; the higher the free radical scavenging activity, the lower the IC_50_ values. For the free radical scavenging activity, IC_50_ is defined as the amount of extract required to reduce the primary radical DPPH˙ concentration by 50%. The results of the anti-oxidant properties are shown in Table 4. Increased concentration of the extracts shows a gradual increase in inactivity. The free radical scavenging activity of *n*-butanol and ethyl acetate extracts of *S. kurdicum* were 48.053±0.04 µg/ml and 48.51±0.09 µg/ml, respectively. The free radical scavenging activity of *n*-butanol and ethyl acetate extracts of *S. asperrimum* were 53.735±0.03 µg/ml and 51.223±0.02 µg/ml, respectively.

***Evaluation of the particle size of liposomes***

The preparation of the liposomal formulation was according to the film hydration method. The formulation ingredients were *n*-butanol extracts of *S. asperrimum*, *S. kurdicum*, lecithin, and cholesterol by the ratio mentioned in Table 5. The liposomes were filtered for size reduction, but only formulation four was filtered because it was smaller than others. The sizes shown in Table 5 are representative of particle sizes before filtration. The particle size of liposomes of formulation 1 was in the micrometer range that could be related to the high content of lecithin. Formulation 2 did not form a homogeny film. The non-homogeny parts developed because of the high content of herbal extract could be observed visually. This might be due to the lower lipid content capacity of particles for forming a homogeny film. Therefore, the optimum formulation of liposomes was formulation four by the ratio of 75 mg lecithin, 25 mg cholesterol, and 50 mg herbal extract. The formulation size was 140 nm, which was detected by a particle size analyzer. 

***Antimicrobial activity***

**Figure 1:**
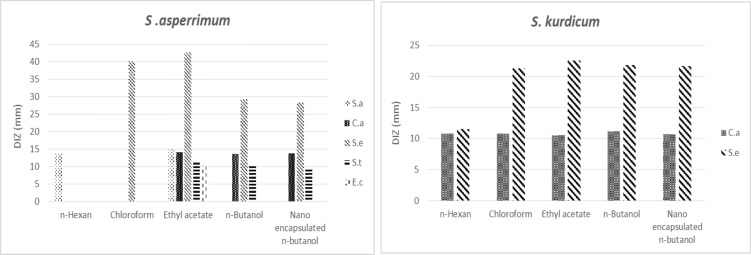
Antimicrobial effects of *Symphytum asperrimum *and *Symphytum kurdicum* extracts against *Escherichia coli***, ***Staphylococcus epidermidis***, ***Staphylococcus aureus***, ***Candida albicans***, ***Salmonella typhi*

The two Gram-positive (*S. aureus* and *S. epidermidis*), two Gram-negative standard bacterial strains (*E. coli* and *S. typhi*), and one fungus (*C. albicans*) have been used. According to the two-way ANOVA test conducted using GraphPad Prism software diameter of the inhibition zone (DIZ) results, ethyl acetate extract of *S. asperrimum* showed significant antibacterial activity against *S. aureus*, *E. coli*, *S. typhi*, and *C. albicans*. Chloroform, ethyl acetate, *n*-butanol, and nanoencapsulated n-butanol extracts of this plant demonstrated significant antibacterial activity against *S. epidermidis* (*P*-value<0.001).* n*-Butanol extract of *S. asperrimum *was effective against *S. typhi* and *C. albicans*. The antimicrobial activity of the different extracts of *S. kurdicum* against *E. coli* and *S. aureus* was not observed, but there are antimicrobial activities against *S. epidermidis*, *S. typhi*, and *C. albicans* (Figure 1). The effects of nanoencapsulated *n*-butanol extract of *S. asperrimum* and *S. kurdicum* were equal to the results of free *n*-butanol extracts and did not improve antimicrobial properties (Table 6).

Additionally, more precise data on the antibacterial activities were obtained by determining bactericidal concentration. The minimum bactericidal concentration (MBC; (mg/ml)) of extracts against the three bacteria is shown in Table 6. The ethyl acetate extract of *S. asperrimum* had the most bactericidal properties against *S. epidermidis*. MBC accredited the extracts antimicrobial activities data.

**Table 1:** GC-MS analysis of the Essential oil content of aerial parts of *Symphytum kurdicum* with their relative retention time, retention index and percentages

NO	Essential oil compounds	Rt	RI STD*	RI	Percentage	Formula
1	Octanal	16.7	979	979	0.3	C_8_H_16_O
2	Nonanal	20.9	1084	1082	0.7	C_9_H_18_O
3	1-Nonanol	23.9	1156	1153	0.7	C_9_H_20_O
4	Decanal	25.1	1186	1184	0.6	C_10_H_20_O
5	1-Decanol	27.8	1255	1254	0.7	C_10_H_22_O
6	Β-Ionone	35.3	1469	1466	1.0	C_13_H_20_O
7	Phenol,2,4-bis(1,1-dimethylethyl)	36.1	1480	1489	0.5	C_14_H_22_O
8	Caryophyllene oxide	38.9	1578	1578	0.7	C_15_H_24_O
9	Hexadecane	39.6	1604	1586	0.9	C_16_H_34_
10	Β-Turmerone	40.7	1637	1637	1.4	C_15_H_22_O
11	1-Decanol,2-methyl	43.3	1720	1721	0.9	C_11_H_24_O
12	Hexafarnesyl acetone	46.3	1827	1829	34.2	C_18_H_30_O
13	6,10,14-trimethylpentadecane-2-ol	46.5	1830	1838	1.4	C18H36O
14	Farnesyl acetone	48.1	1903	1901	0.94	C_18_H_30_O
15	Dibutyl phthalate	48.7	1909	1919	3	C_16_H_22_O_4_
16	Isophytol	49.2	1944	1938	1.01	C_20_H_40_O
17	Eicosane	50.7	2000	1996	0.27	C_20_H_42_
18	Phytol	52.1	2097	2098	33.4	Phytol
19	1-Eicosanol	52.4	2276	2276	1.04	C_20_H_42_O
20	Hentriacontane	57.8	3000	3003	11.4	C_31_H_64_
	Total				95.62	

*Reference(23), Rt: Retention time; RI: Gas chromatographic retention index

**Table 2:** Essential oil content of aerial parts of *Symphytum asperrimum*

NO	Essential oil compounds	Rt	RI STD*	RI	Percentage	Formula
1	Heptane	6.6	960	960	10.3	C_7_H_16_
2	Heptadecane,2,6,10,15-tetramethyl	36.5	1496	1500	3.4	C_21_H_44_
3	Hexadecane	39.5	1604	1597	2.6	C_16_H_34_
4	Β-turmerone	40.7	1637	1637	3.4	C_15_H_22_O
5	Hexafarnesyl acetone	46.3	1827	1828	24.1	C_18_H_36_O
6	Dibutyl phthalate	48.7	1909	1909	4.3	C_16_H_22_O_4_
7	Phytol	53.2	2097	2097	6.0	C_20_H_40_O
8	Hentriacontane	57.7	3000	2993	31.0	C_31_H_64_
	Total				85.35	

*Reference(23), Rt: Retention time; RI: Gas chromatographic retention index

**Table 3:** Total flavonoid and phenol contents of n-butanol and ethyl acetate extracts

	**Total flavonoid** **(**mg Que equivalent/g dried sample)		**Total phenol** **(**mg GA equivalent/g dried sample)	
	***n*** **-Butanol**	**Ethyl acetate**	***n*** **-Butanol**	**Ethyl acetate **
***S. asperrimum***	31.78±0.2	30.34±0.57	57. 3±0.23	52.65 ±7.8
***S. kurdicum***	20.55±1.95	21.62±3.04	42.59±3.01	45.77±2.77

Que: Quercetin; GA: Gallic acid

**Table 4:** Anti-oxidant activity of *Symphytum kurdicum *and *Symphytum asperrimum *extracts by DPPH assay

	**IC**_50_ (ϻg/ml)	
	***n*** **-Butanol extract**	**Ethyl acetate extract**
***S. asperrimum***	48.05±0.04	48.51±0.09
***S. kurdicum***	53.73±0.03	51.22±0.02

**Table 5:** Ratio of lecithin, cholesterol, and an herbal extract for preparation of liposomal formulation

**Number**	**Lecithin (mg)**	**Cholesterol (mg)**	**Herbal extract (mg)**	**Water (ml)**	**Size (nm)**
1	90	30	40	10	1400
2	60	20	100	10	No homogenous film
3	75	25	45	10	600
4	75	25	50	20	450

DIZ: Diameter of the inhibition zone; MBC: Minimum bactericidal concentration

**Table 6:** Comparative assessment of various extracts antimicrobial activity

					**DIZ **(mm)			**MBC **(mg/ml)		
		***E. c***		***S. t***	***S. e***	***C. a***	*** S. a***	***E. c***	***S. e***	***S. a***
***S. asperrimum***	***n*** **-Hexane**	-		-	-	-	13.83±0.85	-	-	25
	**Chloroform**	-		-	40.16±0.62	-	-	-	6.25	-
	**Ethyl acetate**		10.17±0.62	11.33±0.63	42.66±0.85	14.16±0.62	15±0	6.25	3.125	6.25
	***n*** **-Butanol**	-		10.17±0.47	29.33±0.85	13.66±0.23	-	-	6.25	25
	**Nano encapsulated ** ***n*** **-butanol**	-		9.5±0.4	28.33±0.85	13.83±0.23	-	-	-	-
***S. kurdicum***	***n*** **-Hexane**	-		-	11.5±0.4	10.83±0.62	-	-	12.5	25
	**Chloroform**	-		-	21.33±0.47	10.83±0.23	-	-	6.25	25
	**Ethyl acetate**	-		-	22.5±0.4	10.5±0.4	-	-	6.25	25
	***n*** **-Butanol**	-		-	21.83±0.62	11.17±0.23	-	-	6.25	25
	**Nano encapsulated ** ***n*** **-Butanol**	-		-	21.66±0.47	10.66±0.47	-	-	-	-
**Controls**	**DMSO**	-		-	-	-	-	-	-	-
	**Amikacin**	19.5±0.707		ND	ND	ND	ND	ND	ND	ND
	**Tetracycline**	ND		-	39.5±0.707	-	24.5±0.7	ND	ND	ND

DIZ: Diameter of the Inhibition Zone; MBC: Minimum Bactericidal Concentration; E.c: *Escherichia coli*; S.e: *Staphylococcus epidermidis*; S.a: *Staphylococcus aureus*; C.A: *Candida albicans*; S.t: *Salmonella typhi*; ND: Not detected

## Discussion

Several factors, including the type of extraction technique, duration of extraction, temperature, location, soil composition, moisture, and altitude, may influence essential oil components. The difference in the essential oil content between two species of the same genus is predictable, but they may also share many characteristics. The essential oil content of *S. kurdicum* and *S*. *asperrimum* was comparable; however, there is little information on other *Symphytum* species’ essential oil contents from previous studies. A study on the essential oil content of *Glendora rosmarinifolia *yielded similar results (a member of the *Boraginaceae* family). Eicosane, phytol, nonanal, β-ionene, α-pinene, and β-caryophyllene were identified as the main components of the essential oil (24). Eicosane and nonanal were identified in the essential oil compositions of *Cordia* *verbenaceae* (25). Dibutyl phthalate has been reported as the main essential oil constituent of *Anchusa italica* from Iran (26). One of the main components, hentriacontane, one of the main identified compounds in the current study, of the essential oils of the herbs, is a hydrocarbon with antiinflammatory effects, reducing inflammatory mediators (TNF-α, IL-6, PGE2, COX-2, and iNOS), as well as activation of NF-κB and caspase-1 in LPS-stimulated peritoneal macrophages (27). This compound also has an antitumor and cytotoxic effect on lymphoma cells (28). This compound is present in large quantities in both plants so that the essential oils of *S. kurdicum* and *S. asperrimum* could be used as anti-inflammatory agents.

*Symphytum* genus belonging to the *Boraginaceae* family is a well-known herbal medicine with pharmacological potential due to its bioactive components (29). Flavonoids and phenolic derivatives are the significant chemical compounds in the* Symphytum* genus (30). Total phenolic and flavonoid contents in *S. asperrimum* aerial parts extract were significantly higher than those in *S. kurdicum* aerial parts extract. Phytochemical components in the different species are different and could affect their pharmacological activities. Secondary metabolites, including flavonoids and phenolic derivatives, are affected by various factors, such as soil composition, irrigation, and climatic conditions (31, 32). To the best of our knowledge, phenolics and flavonoids have significant beneficial biological activities such as antitumor (33), anti-inflammatory (34), and anti-oxidative activity (35). Phenolics and flavonoids are free radical scavengers due to this facility (36), reducing agents, singlet oxygen, and hydrogen donors (37). Phenolic components inhibit lipid peroxidation and prevent enzyme oxidation (38). In a study, the total phenolic and flavonoid content of the methanol extract of *S. anatolicum* was measured, and the results were 32.7 mg GAE/g and 13.3 mg RE/g (39). The total phenolic and flavonoid contents of aerial parts of *S. asperrimum* and *S. kurdicum* were higher than those of other species in this genus, according to the bibliographic data.

Due to the many side effects and complications initiated by synthetic constituents, there is a demand for natural extracts or essential oils with anti-oxidant activities, particularly those derived from herbs (40). Recent studies show a positive correlation between anti-oxidant activity and total phenol and flavonoid levels (41).* S. asperrimum* and *S. kurdicum *extracts both demonstrated high levels of free-radical-scavenging activity, respectively. The total flavonoid and phenolic contents prove that phenolic components play a significant role in anti-oxidant activity. It is also notable that except phenolics, allantoin, rosmarinic acid, caffeic acid, and their derivatives, as identified in *the Symphytum* family, might be attributed to the high anti-oxidant activity. There have been no previous reports on the anti-oxidant activities of *S. kurdicum* and *S. asperrimum*. At the same time, previous research on other *Symphytum* species, such as *S. officinale*, revealed that its ethanolic extract had high radical scavenging activity against DPPH radicals (39.97 µg/ml) in comparison with an aqueous extract (96.21 µg/ml) (35). The free radical scavenging activities of methanol and ethyl acetate extracts of *S. anatolicum* were 2.70 ± 0.15 mg/ml and 10.57 ± 0.01 mg/ml (42). According to the reports other *Symphytum* species that have also shown significant anti-oxidant properties (43, 44). The potent anti-oxidant properties could be attributed to their phenol and flavonoid contents, confirming that phenolic compounds like caffeic acid derivatives play a prominent role in anti-oxidant activity (39). 

The *in vitro* antibacterial properties of these two Iranian native plants have been investigated for the first time. According to the results of the DIZ and MBC values, *S. epidermidis* was the most sensitive microorganism. One of the most influential herbal families with antimicrobial activities is the *Boraginaceae* (45). Previous research tested the inhibitory effects of methanolic extract of the *S. officinale L*. leaves against *S. aureus*, *Pseudomonas aeruginosa*, *Salmonella typhimurium*; *Shigella sonnei*, *Klebsiella pneumonia*, and *Escherichia coli*. The antibacterial test results revealed that *S. officinal*e L. extract had antibacterial activity against the tested strains. DIZ was greater than 7 mm (46). The strong antimicrobial activities of the extracts related to the phytochemicals of the *Symphytum* genus, including phenolic compounds (caffeic acid, allantoin, and luteolin glycoside), are possibly related to the synergetic effects of compounds, as well as to other bioactive components existing in the whole parts of the plant (3). Mangonia *et al*. investigated the *in* *vitro* and *in* *vivo* characteristics of phycocyanin liposomal formulation. The liposome structure was composed of soy phosphatidylcholine and cholesterol, and results showed that the preparation method could affect the liposome characteristics. According to their findings, the liposomal formulation was an excellent choice for topical formulation containing phycocyanin as an anti-inflammatory agent, and encapsulation could increase the anti-inflammatory effect dose-dependently. Therefore, a lower concentration of liposomal formulation had an impact equal to the higher concentration of free phycocyanin (47). Aisha *et al*. evaluated the liposomal formulation of *Orthosiphon stamineus* extracts containing soybean phospholipids.

Liposome structure could increase the ethanolic extract solubility about four times, and the liposome entrapment efficiency reached 66%. Evaluation of the intestinal absorption showed significant enhancement that could result from higher aqueous solubility of the liposomal extract. The mentioned formulation could be a choice for oral or topical drug delivery (48). Matouskova *et al*. examined the antimicrobial activities of several herbal extracts encapsulated in different particles and compared the results with non-encapsulated extracts. The prepared particles were stable, and the antimicrobial effect was evaluated against four bacterial strains. The stability of liposomes was higher than chitosan particles, but the inhibitory effect of chitosan particles was very high (49). In contrast to previous studies, the liposomal extract’s antimicrobial effect was lower than the free form in the present study. As the liposome contains a bilayer structure, the membrane will lead to the sustainable release of the loaded component that can be a reason for the lower effect of the liposomal form, but confirmation needs further studies.

## Conclusion

In this study, the most abundant constituent of both essential oil compositions with beneficial biological effects was a hydrocarbon compound, hentriacontane. Significant anti-oxidant and antimicrobial activities of *n*-butanol and ethyl acetate extracts were mainly attributed to the high levels of phenolic and flavonoid constituents of* S. asperrimum* and *S. kurdicum*. The extracts were encapsulated in the liposomal formulation using the film hydration method, and the size of final liposomes was in the nanometer range; the result of liposomes antimicrobial activity was in conflict with previous studies, and the probable reason might be the existence of bilayer structure of liposome which retards the release of the extract. Further studies should be conducted to confirm the results of the present study. *In vivo* assessment of anti-oxidant and antimicrobial properties besides the toxicity studies of the extracts of *S. asperrimum and S. kurdicum* are consequently suggested.

## Authors’ Contributions

EM, HN, FKH, and SM Conceived the study; EM, HN, HV, FKH, SM, and SH Collected and processed the data and performed experiments; EM, SM, SH, HN, and FKH Analyzed the data and prepared the draft manuscript; HN, SH, and EM Critically revised the paper; HN, HV, and SH Supervised the research; EM, HN, HV, FKH, SM, and SH Approved the final version to be published.

## Conflicts of Interest

The authors declare that they have no conflicts of interest.

## References

[1] Bilal M, Rasheed T, Iqbal HMN, Hu H, Wang W, Zhang X (2017). Macromolecular agents with antimicrobial potentialities: A drive to combat antimicrobial resistance. Int J Biol Macromol.

[2] Sirbu R, Gabriela S, Tomescu A, Ionescu A, Cadar E (2019). Evaluation of antioxidant and antimicrobial activity in relation to total phenolic content of green algae from black sea. Revista de Chimie.

[3] Salehi B, Sharopov F, Boyunegmez Tumer T, Ozleyen A, Rodríguez-Pérez C, Ezzat SM (2019). Symphytum species: A comprehensive review on chemical composition, food applications and phytopharmacology. Molecules.

[4] Mohan A, Alzubaidy A (2018). A comparative systematic study of the genus Symphytum l (Boraginaceae) with new first record of the species Symphytum tuberosum l. From iraQ. Plant Arch.

[5] Nastić N, Borrás-Linares I, Lozano-Sánchez J, Švarc-Gajić J, Segura-Carretero A (2020). Comparative assessment of phytochemical profiles of comfrey (Symphytum officinale L ) root extracts obtained by different extraction techniques. Molecules (Basel, Switzerland).

[6] Karimov V, Alizada V, Mehdiyeva N (2018). Useful features of the species of Boraginaceae juss. Family spread in azerbaijan. Web of Scholar.

[7] Rocha R, da Luz DE, Engels C, Pileggi SA, de Souza Jaccoud Filho D, Matiello RR (2009). Selection of endophytic fungi from comfrey (Symphytum officinale ) for in vitro biological control of the phytopathogen Sclerotinia sclerotiorum (Lib ). Braz J Microbiol.

[8] Staiger C (2013). Comfrey root: from tradition to modern clinical trials. Wien Med Wochenschr Suppl.

[9] Knaak N, da Silva LD, Andreis TF, Fiuza LM (2013). Chemical characterization and anti-fungal activity of plant extracts and essential oils on the Bipolaris oryzae and Gerlachia oryzae phytopathogens. Australas Plant Pathol.

[10] Svetlichny G, Külkamp-Guerreiro IC, Dalla Lana DF, Bianchin MD, Pohlmann AR, Fuentefria AM (2017). Assessing the performance of copaiba oil and allantoin nanoparticles on multidrug-resistant Candida parapsilosis. J Drug Deliv Sci Technol.

[11] Le V, Dolganyuk V, Sukhikh A, Babich O, Ivanova S, Prosekov A (2021). Phytochemical analysis of Symphytum officinale root culture extract. Appl Sci.

[12] Danafar H, Davaran S, Rostamizadeh K, Valizadeh H, Hamidi M (2014). Biodegradable m-PEG/PCL Core-Shell Micelles: preparation and characterization as a sustained release formulation for curcumin. Adv Pharm Bull.

[13] Kamran U, Bhatti HN, Iqbal M, Jamil S, Zahid M (2019). Biogenic synthesis, characterization and investigation of photocatalytic and antimicrobial activity of manganese nanoparticles synthesized from Cinnamomum verum bark extract. J Mol Struct.

[14] Singh A, Vengurlekar P, Rathod S (2014). Design, development and characterization of liposomal neem gel. Int J Pharm Sci Res.

[15] Tometri SS, Ahmady M, Ariaii P, Soltani MS (2020). Extraction and encapsulation of Laurus nobilis leaf extract with nano-liposome and its effect on oxidative, microbial, bacterial and sensory properties of minced beef. J Food Meas Charact.

[16] Zengin G, Sinan KI, Ak G, Angeloni S, Maggi F, Caprioli G (2021). Preliminary investigation on chemical composition and bioactivity of differently obtained extracts from Symphytum aintabicum Hub Mor &Wickens. Biochem Syst Ecol.

[17] Hamedeyazdan S, Fatemeh F, Asnaashari S (2013). Chemical composition of the essential oil from Marrubium persicum C A Mey. (Lamiaceae). Pharm Sci.

[18] Ghasemian-Yadegari J, Hamedeyazdan S, Nazemiyeh H, Fathiazad F (2019). Evaluation of phytochemical, antioxidant and antibacterial activity on Astragalus Chrysostachys Boiss. Roots. Iran J Pharm Res.

[19] Hamedeyazdan S, Sharifi S, Nazemiyeh H, Fathiazad F (2014). Evaluating antiproliferative and antioxidant activity of Marrubium crassidens. Adv Pharm Bull.

[20] Fathiazad F, Mazandarani M, Hamedeyazdan S (2011). Phytochemical analysis and antioxidant activity of Hyssopus officinalis L from Iran. Adv Pharm Bull.

[21] Plangsombat N, Rungsardthong K, Kongkaneramit L, Waranuch N, Sarisuta N (2016). Anti-inflammatory activity of liposomes of Asparagus racemosus root extracts prepared by various methods. Exp Ther Med.

[22] Gortzi O, Lalas S, Chinou I, Tsaknis JoI (2007). Reevaluation of bioactivity and antioxidant activity of Myrtus communis extract before and after encapsulation in liposomes. Eur Food Res Technol.

[23] Babushok V, Linstrom P, Zenkevich I (2011). Retention indices for frequently reported compounds of plant essential oils. J Phys Chem Ref Data.

[24] Poma P, Labbozzetta M, Notarbartolo M, Bruno M, Maggio A, Rosselli S (2018). Chemical composition, in vitro antitumor and pro-oxidant activities of Glandora rosmarinifolia (Boraginaceae) essential oil. PloS one.

[25] Meccia G, Rojas LB, Velasco J, Díaz T, Usubillaga A, Arzola JC (2009). Chemical composition and antibacterial activity of the essential oil of cordia verbenacea from the Venezuelan andes. Nat Prod Commun.

[26] Kazemi M (2013). Essential oil composition of Anchusa italica from Iran. Chem Nat Compd.

[27] Kim SJ, Chung WS, Kim SS, Ko SG, Um JY (2011). Antiinflammatory effect of Oldenlandia diffusa and its constituent, hentriacontane, through suppression of caspase-1 activation in mouse peritoneal macrophages. Phytother Res.

[28] Quintanilla-Licea R, Morado-Castillo R, Gomez-Flores R, Laatsch H, Verde-Star MJ, Hernández-Martínez H (2012). Bioassay-guided isolation and identification of cytotoxic compounds from Gymnosperma glutinosum leaves. Molecules.

[29] Paun G, Neagu E, Moroeanu V, Ionescu E, Radu G (2016). Phytochemical analysis and biological activity of the phenolic rich extract of Impatiens noli-tangere and Symphytum officinalis. Planta Medica.

[30] Varvouni E-F, Zengin G, Graikou K, Ganos C, Mroczek T, Chinou I (2020). Phytochemical analysis and biological evaluation of the aerial parts from Symphytum anatolicum Bois and Cynoglottis barrelieri (All Vural & Kit Tan (Boraginaceae). Biochem Syst Ecol.

[31] Gharib A, Godarzee M (2016). Determination of secondary metabolites and antioxidant activity of some Boraginaceae species growing in Iran. Trop J Pharm Res.

[32] Chandra S, Khan S, Avula B, Lata H, Yang MH, Elsohly MA (2014). Assessment of total phenolic and flavonoid content, antioxidant properties, and yield of aeroponically and conventionally grown leafy vegetables and fruit crops: a comparative study. Evid Based Complement Alternat Med.

[33] Shahidi F, Yeo J (2018). Bioactivities of phenolics by focusing on suppression of chronic diseases: A Review. Int J Mol Sci.

[34] Vostinaru O, Conea S, Mogosan C, Toma C, Borza C, Vlase L (2018). Anti-inflammatory and antinociceptive effect of Symphytum officinale root. Rom Biotechnol Lett.

[35] Üstün Alkan F, Anlas C, Ustuner O, Bakırel T, Sari A (2014). Antioxidant and proliferative effects of aqueous and ethanolic extracts of Symphytum officinale on 3T3 Swiss albino mouse fibroblast cell line. Asian J Plant Sci.

[36] Amic D, Davidović-Amić D, Beslo D, Trinajstić N (2003). Structure-radical scavenging activity relationships of flavonoids. Croat Chem Acta.

[37] Ghasemzadeh A, Ghasemzadeh N (2011). Flavonoids and phenolic acids: Role and biochemical activity in plants and human. J Med Plant Res.

[38] Maqsood S, Benjakul S, Abushelaibi A, Alam A (2014). Phenolic compounds and plant phenolic extracts as natural antioxidants in prevention of lipid oxidation in seafood: a detailed review. Compr Rev Food Sci Food Saf.

[39] Elisavet-Foteini V (2020). Phytochemical analysis and biological evaluation of the aerial parts from Symphytum anatolicum Boiss and Cynoglottis barrelieri (All ) Vural & Kit Tan (Boraginaceae). Biochem Syst Ecol.

[40] Sevgi K, Tepe B, Sarikurkcu C (2015). Antioxidant and DNA damage protection potentials of selected phenolic acids. Food Chem Toxicol.

[41] Aryal S, Baniya MK, Danekhu K, Kunwar P, Gurung R, Koirala N (2019). Total phenolic content, flavonoid content and antioxidant potential of wild vegetables from Western Nepal. Plants.

[42] Ozer S, Tlili N (2019). LC-ESI-MS/MS characterization of phytochemical and enzyme inhibitory effects of different solvent extract of Symphytum anatolicum. Ind Crops Prod.

[43] Trifan A, Opitz SEW, Josuran R, Grubelnik A, Esslinger N, Peter S (2018). Is comfrey root more than toxic pyrrolizidine alkaloids? Salvianolic acids among antioxidant polyphenols in comfrey (Symphytum officinale L ) roots. Food Chem Toxicol.

[44] Vergun O, Brindza J, Rakhmetov D (2017). Total antioxidant activity of plants of Symphytum L species. Agrobiodivers Improv Nutr Health Life Qual.

[45] Tufa T, Damianakos H, Graikou K, Chinou L (2017). Comparative study of naphthoquinone contents of selected greek endemic Boraginaceae plants - antimicrobial activities. Nat Prod Commun.

[46] Bouzada M, Fabri R, Nogueira M, Konno T, Garcia G, Scio E (2009). Antibacterial, cytotoxic and phytochemical screening of some traditional medicinal plants in Brazil. Pharm Biol.

[47] Manconia M, Pendás J, Ledón N, Moreira T, Sinico C, Saso L (2009). Phycocyanin liposomes for topical anti-inflammatory activity: in-vitro in-vivo studies. J Pharm Pharmacol.

[48] Aisha AF, Majid AM, Ismail Z (2014). Preparation and characterization of nano liposomes of Orthosiphon stamineus ethanolic extract in soybean phospholipids. BMC Biotechnol.

[49] Matouskova P, Marova I, Bokrova J, Benesova P (2016). Effect of encapsulation on antimicrobial activity of herbal extracts with lysozyme. Food Technol Biotechnol.

